# 晚期非小细胞肺癌*ALK*融合基因及其他少见驱动基因阳性靶向治疗进展

**DOI:** 10.3779/j.issn.1009-3419.2017.01.10

**Published:** 2017-01-20

**Authors:** 权 张, 树才 张

**Affiliations:** 101149 北京，首都医科大学附属北京胸科医院肿瘤内科 Department of Medical Oncology, Beijing Chest Hospital, Capital Medical University, Beijing Tuberculosis and Thoracic Tumor Research Institute, Beijing 101149, China

**Keywords:** 肺肿瘤, 靶向治疗, ALK, Lung neoplasms, Targeted therapy, ALK

## Abstract

靶向治疗是驱动基因阳性的晚期非小细胞肺癌重要的治疗手段之一，这个领域的治疗进展日新月异，包括新靶点的发现，新药物的问世以及靶向联合治疗方案的应用等，同时研究人员也深入地研究了各种靶向药物原发或获得性耐药机制。本综述旨在总结间变淋巴瘤激酶融合基因（anaplastic lymphoma kinase, *ALK*）及其他少见驱动基因阳性的非小细胞肺癌靶向治疗的新进展。

## Abstract (en)

Targeted therapy was one of the major treatments in advanced non-small cell lung cancer (NSCLC) with positive driver genes. This area of research progresses day by day, with novel target discoveries, novel drug development, and use of novel combination treatments. Researchers have also undergone deep investigation about the molecular mechanisms underlying inherent or acquired resistance to these targeted therapies. This review aimed to summarize the advanced developments of targeted therapy for anaplastic lymphoma kinase (ALK) and other rare driver genes in NSCLC.

肺癌在我国恶性肿瘤发病率及死亡率均居首位，在初诊时70%的患者已错失手术治疗的机会，更依赖于传统放化疗、靶向治疗以及免疫治疗等手段^[1]^。随着分子检测技术的不断更新及靶向治疗新药的陆续问世，越来越多的驱动基因得以被识别，并可有相应的靶向药物可供选择。本文针对2016年晚期非小细胞肺癌（non-small cell lung cancer, NSCLC）间变淋巴瘤激酶融合基因（anaplastic lymphoma kinase, *ALK*）阳性及其他少见驱动基因的靶向治疗进展作一综述。

## 1 棘皮动物微管相关蛋白4-间变淋巴瘤激酶融合基因（echinoderm microtubule associated protein-like 4-ALK, EML4-ALK）

2007年Soda等^[2]^在NSCLC患者的组织标本中报道了*EML4*-*ALK*融合基因。近些年多个ALK抑制剂的相继问世，同时针对ALK抑制剂的耐药机制研究也越来越深入。

### 1.1 一代ALK抑制剂

2011年克唑替尼作为全球首个ALK抑制剂获得美国食品药品管理局（Food and Drug Administration, FDA）批准，用于治疗ALK阳性的局部晚期或转移性NSCLC患者。PROFILE1007^[3]^、PROFILE1014^[4]^临床研究结果证实了无论是在二线还是在一线应用克唑替尼，在疗效和安全性方面均较化疗有明显优势；2016年美国临床肿瘤学会（American Society of Clinical Oncology, ASCO）公布了PROFILE1029研究^[5]^的初步结果，入组人群为东亚非鳞癌患者，对比了一线应用克唑替尼与培美曲塞联合铂类药物化疗的疗效、安全性的差异，结果进一步证实了PROFILE1014的结论，克唑替尼组与化疗组客观缓解率（objective response rate, ORR）分别为88%和46%（*P* < 0.01），无进展生存期（progression-free survival, PFS）分别为11.1个月和6.8个月（*P* < 0.01）。基于这一系列临床试验的结果，无论是美国国立综合癌症网络（National Comprehensive Cancer Network, NCCN）指南^[6]^还是中国表皮生长因子受体（epidermal growth factor receptor, *EGFR*）基因敏感性突变和ALK阳性NSCLC诊断治疗指南^[7]^针对ALK阳性的局部晚期或转移性NSCLC患者均推荐一线治疗首选克唑替尼。

### 1.2 二代ALK抑制剂

色瑞替尼（Ceritinib）作为一种二代ALK抑制剂，ASCEND-1^[8, 9]^结果显示，既往接受或未接受过克唑替尼治疗的晚期NSCLC患者，接受Ceritinib治疗，ORR为58%，PFS为7个月，其中接受过克唑替尼治疗后进展的人群，ORR为56%，PFS为6.9个月，未经克唑替尼治疗的患者PFS为18.4个月。2014年4月29日FDA批准了Ceritinib用于克唑替尼治疗进展后或对克唑替尼不能耐受的晚期NSCLC患者。2016年WCLC公布了Ⅲ期随机多中心研究ASCEND-4^[10]^的结果，该研究在初治ALK阳性NSCLC中对比了一线应用Ceritinib同化疗之间疗效的差异，入组376例患者，Ceritinib组ORR为72.7%，PFS长达16.6个月，不伴有脑转移的患者中位PFS长达26.3个月，化疗组ORR仅为26.7%，PFS为8.1个月。此研究使Ceritinib有望成为ALK阳性NSCLC患者一线治疗的新选择。

2014年7月，日本批准了一种新的第二代ALK抑制剂-Alectinib，北美Ⅱ期（NP28671）^[11]^和全球Ⅱ期（NP28673）^[12]^临床研究结果显示ORR分别为52%和50%，PFS分别为8.1个月和8.9个月，耐受性良好，2015年FDA基于上述两项研究批准Alectinib用于经治的ALK阳性NSCLC。关于Alectinib对比克唑替尼治疗初治ALK阳性NSCLC患者的Ⅲ期临床研究（J-ALEX），2016 ASCO^[13]^也有了初步结果，ORR分别为85.4%和70.2%，Alectinib组中位PFS尚未达到，克唑替尼组中位PFS为10.2个月，安全性方面，Alectinib组耐受性更好，因任何原因AE停药率为8.7%，而克唑替尼组停药率达20.2%，无论疗效及安全性方面，Alectinib组均有更大优势，Alectinib具有成为ALK阳性NSCLC患者一线标准治疗的潜力，但本研究两组入组基线稍欠平衡（克唑替尼组基线脑转移患者更多），且仅为日本Ⅲ期临床研究数据，其结果还有待全球ALEX结果证实。

2016年ASCO公布了关于二代ALK抑制剂-Brigatinib的国际多中心Ⅱ期研究（ALTA）结果^[14]^，入组人群均为克唑替尼耐药的ALK阳性NSCLC患者，90 mg剂量组中位PFS达9.2个月，180 mg剂量组中位PFS已超过1年，达12.9个月，2个剂量组疗效确切，安全性可耐受，疗效和安全性支持180 mg方案，Brigatinib有望成为治疗克唑替尼耐药的ALK阳性NSCLC的新选择。目前Brigatinib 180 mg方案对比克唑替尼治疗未接受过ALK抑制剂治疗患者的Ⅲ期临床研究已经启动（ALTA-1L, NCT02737501）。

### 1.3 三代ALK抑制剂

Lorlatinib是辉瑞公司推出的第三代ALK抑制剂，2016年ASCO公布的Ⅰ期/Ⅱ期临床研究数据^[15]^显示其出色的疗效，入组41例ALK阳性NSCLC患者，14例患者接受过一种ALK抑制剂治疗，26例患者接受过1种以上的ALK抑制剂资料，两组ORR分别为57%和42%，PFS分别为13.5个月和9.2个月，整体ORR为46%，PFS为11.4个月。

### 1.4 ALK抑制剂耐药机制

目前ALK抑制剂继发耐药^[16-18]^机制大致分为几类，一类为ALK继发耐药突变，其中又可分为ALK激酶区突变和*ALK*基因拷贝数扩增。ALK激酶区突变常包括L1196M、L1152R、G1202R、G1269A、1151Tins、S1206Y、C1156Y、F1174C、D1203N等。也称为“ALK主导机制”参与获得性耐药。还有一类为“ALK非主导机制”参与的获得性耐药，主要包括其他致癌驱动基因的活化，或者通过旁路引起下游信号通路的再激活，常见的是*EGFR*突变或磷酸化、*KRAS*突变和*c*-*KIT*扩增。同时肿瘤异质性的存在也增加了耐药问题的复杂性，同一肿瘤可见多个驱动基因、多个激酶区突变或多个耐药机制的共存现象。另外仍然有部分患者获得性耐药的机制尚不清楚。

### 1.5 ALK抑制剂耐药后治疗策略

针对不同的耐药原因，采取不同的治疗方法。“ALK主导机制”导致的获得性耐药，可通过二代、三代ALK抑制剂来治疗，对于常见的L1196M、G1269A、C1156Y、S1206Y、F1174L突变，Ceritinib、Alectinib、Brigatinib、Lorlatinib均可克服，然而Ceritinib对G1202R、1151Tins、F1174C、L1198F耐药，Alectinib对G1202R、V1180L、I1171T、1151Tins突变耐药，Brigatinib对于G1202R、1151Tins突变需治疗加量^[14]^，三代药物Lorlatinib可克服常见克唑替尼耐药突变^[15]^，对V1180L、I1171T突变效果不详，然而对于L1198F耐药突变，克唑替尼却可克服L1198F耐药^[19]^。因此ALK耐药后明确耐药突变的类型对于后续药物的选择仍然有一定的临床意义。而“ALK非主导机制”导致的获得性耐药可通过联合热休克蛋白、其他特定靶点的靶向药物、化疗等办法来克服。

## 2 *ROS1*基因重排

2007年Rikova等^[20]^首次在NSCLC中发现*ROS1*基因重排，目前已发现*SLC34A2*-*ROS1*、*CD74*-*ROS1*、*TPM3*-*ROS1*、*SDC4*-*ROS1*、*EZR*-*ROS1*、*FIG*-*ROS1*等多种融合基因^[21]^，其中CD74-ROS1最为常见，可通过FISH、RT-PCR等方法检测该融合基因的存在，在NSCLC中*ROS1*重排检出率为1%-2%，多见于不吸烟或轻度吸烟的年轻患者中。

### 2.1 ROS1抑制剂

Bergethon等^[22]^最先报道了应用克唑替尼治疗ROS1阳性NSCLC患者的成功案例。2014年Shaw等^[23]^公布了一项克唑替尼用于ROS1阳性的Ⅰ期临床研究（NCT00585195），结果显示有效率为72%，中位缓解时间17.6个月，PFS为19.2个月，常见副作用较前相似，多见视觉障碍、腹泻、恶心及水肿等。2015年4月FDA授予克唑替尼治疗ROS1阳性NSCLC的突破性药物资格，并于2016年3月批准克唑替尼用于ROS1阳性NSCLC的治疗。目前应用克唑替尼治疗东亚ROS1阳性NSCLC患者的Ⅱ期临床研究（NCT01945021）^[24]^仍在进行中，初步结果显示ORR为69.3%，PFS为13.4个月。2016年欧洲肿瘤内科学会（European Society for Medical Oncology, ESMO）报道了一项来自韩国应用Ceritinib治疗*ROS1*重排阳性NSCLC的Ⅱ期临床研究（NCT01964157）^[25]^，结果显示28例可评估患者中ORR为60.7%，32例接受Ceritinib治疗的患者中位PFS为10.0个月，进一步证实了Ceritinib用于治疗*ROS1*重排阳性NSCLC的有效性。其中由于ALK和ROS1的激酶活性区域有70%的相似性，因此ALK抑制剂很多是可以用于ROS1阳性治疗的。基础研究显示克唑替尼、Ceritinib、Brigatinib对CD74 -ROS1的抑制效果很不错，但Alectinib却没有显示出具有抑制活性，因此不能直接应用到ROS1阳性的患者上，最起码不能应用到CD74-ROS1的融合突变的患者上。第三代ALK抑制剂Lorlatinib同样也是一个强效ROS1抑制剂，2016年ASCO公布的Ⅰ期/Ⅱ期临床研究数据^[15]^显示入组12例ROS1阳性NSCLC患者，其中2例曾接受ROS1抑制剂治疗以及4例未曾接受ROS1抑制剂治疗的患者靶病灶疗效均达PR，疗效显著。

### 2.2 ROS1抑制剂耐药

ROS1抑制剂耐药常由激酶区域的二次突变所导致^[26, 27]^，常见有CD74-ROS1的G2032R突变，其他还包括L2026M、G2101A、K2003I、L2155S、L1951R等，G2032R突变影响了克唑替尼与ROS1的结合，导致克唑替尼耐药，同样有研究者利用分子对接模拟发现Ceritinib对G2032R的亲和力要比野生型ROS1弱5倍-10倍，G2032R突变也可导致Ceritinib耐药。其他耐药途径有旁路激活，如*c*-*KIT*或*KRAS*基因突变等，EGFR的代偿性高表达，钙粘附蛋白E表达量丢失，波形蛋白和人纤维连接蛋白表达增加等。

### 2.3 ROS1抑制剂耐药策略

体外实验^[28]^表明Lorlatinib对于*ROS1*基因上的G2032R突变、L2026M突变具有抑制效果，针对G2032R突变细胞系半抑制浓度（half maximal inhibitory concentration, IC_50_）值不足克唑替尼及Ceritinib的十分之一。在动物模型的体内实验上，Lorlatinib对于FIG-ROS1、CD74-ROS1和存在G2032R耐药突变的CD74-ROS1都具有抑制活性。Lorlatinib有望克服G2032R突变导致的ROS1抑制剂耐药问题。同时基础研究还显示ROS1阳性克唑替尼耐药后的G2101A、L2026M和G2032R的肿瘤细胞系对Foretinib（GSK1363089）敏感，但是L2155S对于Foretinib仍然耐药。ROS1阳性克唑替尼耐药后L2026M、L1951R和G2032R对卡博替尼（XL184）敏感，可以被卡博替尼（XL184）克服^[29]^。因此，针对不同的耐药突变情况可能需要选择不同的药物，同时针对激酶区域二次突变以外的抗耐药策略，需根据具体情况给予针对性的治疗方法，比如，在EGFR高表达时尝试同时使用EGFR的靶向药物吉非替尼或西妥昔单抗联合克唑替尼。目前上述大部分研究多为基础研究探索，仍需相关临床实践进一步证实。

## 3 MET

c-MET属于受体酪氨酸蛋白激酶家族成员，HGF是其特异性的配体，当c-MET与配体HGF结合后，胞质中酪氨酸残基发生磷酸化，激活酪氨酸激酶，激活PI3K-AKT、RAS-MAPK、STAT3等多个信号传导通路，参与肿瘤侵袭、转移等过程。同时c-MET亦可不依赖HGF实现信号通路激活，包括*MET*扩增、MET过表达、突变以及14外显子跳跃、重排等^[30-32]^。近两年ASCO相继报道了对于原发性MET高扩增或过表达的NSCLC患者克唑替尼治疗ORR达46%-67%，EGFR-TKI耐药后出现获得性MET过表达时，在不合并T790M突变存在时克唑替尼治疗有效。2016年ASCO中吴一龙等^[33]^报道了MET抑制剂capmatinib（INC280）联合吉非替尼治疗EGFR-TKI耐药后MET阳性NSCLC的临床疗效（NCT01610336），结果显示ORR为31%，疾病控制率（disease control rate, DCR）为81%，*c*-*MET*基因拷贝数高扩增组ORR为50%，DCR为84%。MET14外显子跳跃，2014年由美国癌症基因研究组（Cancer Genome Atlas, TCGA）首次报道，肺腺癌中发生率为3%-4%，多见于腺鳞癌和肉瘤样癌。MET14外显子编码的结构域为MET的负性调控区，参与MET蛋白的泛素化及降解，MET14外显子跳跃后可导致MET蛋白泛素化及降解减低，增加MET蛋白的稳定性，导致下游信号的激活^[34]^。PROFILE1001研究（NCT00585195）^[35]^纳入了21例MET 14外显子跳跃的NSCLC患者，接受克唑替尼治疗后，18例可评估病例ORR为44%，DCR为94%，PFS结果仍有待继续随访。

## 4 RET

RET是神经胶质细胞驱动神经营养因子家族的细胞外信号分子或配体，RET原癌基因/甲状腺乳头样癌基因重排首先在甲状腺乳头状癌中被发现，并被证实其致癌性。2012年Ju等^[36]^首次在肺腺癌中报道了*KIF5B*-*RET*融合基因的存在，其后相继有研究者^[37, 38]^在肺腺癌组织中发现CCDC6-RET、NCOA4-RET等*RET*融合基因亚型，可通过RT-PCR、FISH、IHC等方法检测，在NSCLC中*RET*融合检出率为1%左右，也多见于不吸烟、年轻患者中。RET为索拉菲尼、凡德他尼、卡博替尼等多靶点药物的治疗靶点之一，上述药物均不是专一用于RET，其用于治疗RET阳性的NSCLC的效果也有待进一步评价。近两年有多项在肺癌中开展的相关研究结果公布，2015年ASCO公布了卡博替尼治疗晚期RET阳性肺腺癌的Ⅱ期临床研究结果，入组16例患者，ORR为38%，PFS为7个月，总生存期（overall survival, OS）为10个月。2016年ASCO公布了LURET研究结果^[39]^，该研究为凡德他尼治疗晚期*RET*重排NSCLC的Ⅱ期、开放性、单臂研究，结果显示非鳞癌RET阳性率为2%，入组19例患者，KIF5B-RET、CCDC6-RET及未知亚型分别为10例、6例及3例，ORR分别为20%、83%和67%，中位PFS分别为2.9个月、8.3个月和4.7个月，进一步肯定了凡德他尼在*RET*重排NSCLC中的抗肿瘤作用，同时研究结果提示凡德他尼可能对CCDC6-RET亚型效果更佳。Velcheti等^[40]^于2016年ESMO公布了Lenvatinib用于*RET*融合基因阳性肺腺癌患者的Ⅱ期临床研究结果，入组25例患者，ORR为16%，中位PFS为7.3个月，其中KIF5B-RET和其他亚型ORR分别为15.4%和16.7%，中位PFS为3.6个月和9.1个月，既往接受RET靶向治疗（凡德他尼或卡博替尼）与未接受RET靶向治疗分别为7例和18例，ORR分别为14.3%和16.7%，疗效结果相似，Lenvatinib可作为凡德他尼或卡博替尼耐药后的治疗选择之一。

## 5 HER2

HER2通过活化PI3K-AKT、MEK-ERK等信号通路，促进肿瘤细胞增殖，主要机制包括*HER2*基因的扩增、HER2蛋白过表达和HER2酪氨酸激酶结构域的突变。对于HER2过表达的NSCLC患者，无论是单用化疗、还是单一曲妥珠单抗治疗或曲妥珠单抗治疗联合全身化疗均未显示出显著的临床疗效^[41-44]^。HER2扩增是NSCLC中EGFR-TKI耐药的机制之一，而HER2外显子20突变相对于过表达或扩增与肺癌的致病机制更加相关。早在2006年Cappuzzo等^[45]^报道了外显子20突变的存在，应用曲妥珠单抗和紫杉醇化疗，疗效可达部分缓解。Mazieres等^[46]^报道了65例HER2外显子20突变病例，其中22例在传统化疗后接受抗HER2治疗，ORR达50%，DCR达82%，PFS达5.1个月，其中曲妥珠单抗治疗组（*n*=15）DCR达93%，阿法替尼组（*n*=3）DCR为100%。2016年Ohashi等^[47]^开展了一项针对T-DM1在复发或转移的HER2阳性NSCLC中疗效的非随机开放性多中心Ⅱ期临床研究，有待相关临床结果的公布。同时Ou等^[48]^ 还发现肺腺癌HER2跨膜区突变（V659/G660）也是罕见的驱动基因之一，而且对 Afatinib治疗疗效显著（4例接受 Afatinib治疗的患者有3例获得临床缓解）。2016年由Eng等^[49]^公布了一组来自斯隆凯特纪念医院的回顾性资料，分析了64例*HER2*突变的转移性NSCLC治疗结果，化疗组治疗时间较HER2靶向治疗组（包括Dacomitinib、Afatinib、Neratinib、Lapatinib）更长，HER2靶向治疗组大部分患者没有获益或者获益时间很短，化疗仍是该类患者的标准治疗，需要更多的研究去确认针对*HER2*突变的转移性NSCLC更为有效的靶向治疗药物。

## 6 BRAF

*RAF*基因家族由BRAF、ARAF和CRAF组成，是RAF-MEK-ERK信号传导途径的重要环节，其中BRAF激酶是该途径的最强激活剂，激活多种细胞因子，调控细胞增殖、分化和凋亡。NSCLC中*BRAF*突变占1%-5%，存在多种突变位点^[50]^，包括V600E、G469A、D594G等，来自亚洲的数据^[51, 52]^显示突变率相对白种人较低，突变类型以V600E为主。2015年Hyman等^[53]^及Gautschi等^[54]^临床研究结果显示Vemurafenib在*BRAF* V600E突变的非黑色素瘤及*BRAF*突变的NSCLC中显著的临床疗效。2016年ASCO公布一项达拉非尼联合曲美替尼治疗复治*BRAF* V600E突变的晚期NSCLC的开放的Ⅱ期临床研究结果^[55]^，结果显示ORR达63%，DCR达79%，初步PFS已达9.7个月，而达拉非尼单药治疗初治或复治*BRAF* V600E突变的晚期NSCLC单臂多中心Ⅱ期研究结果^[56]^显示ORR、DCR分别仅为33%和58%，PFS仅有5.5个月。

## 7 结语

随着制药业的快速发展，各种临床试验的开展，以及分子生物学技术的更新，更多的驱动基因得以被识别，不断涌现新的靶向药物，并得以快速应用于临床，改善临床疗效，减轻副作用，同时随着靶向药物耐药机制的研究更为深入，耐药后的临床处理方式也越来越成熟，肺癌的治疗必将实现真正的个体化，但少见驱动基因客观上发生率低，相对病例数较少，也需要大家更多的关注、合作以及更为深入的研究，让更多的患者临床获益。
